# Advancing Digital Twin-Based Collision Avoidance: A Comprehensive Analysis of Communication Networks for Safety-Critical Applications in Industry 4.0 †

**DOI:** 10.3390/s24051405

**Published:** 2024-02-22

**Authors:** Christian Moldovan, Silas Ulrich, Volker Köster, Janis Tiemann, Andreas Lewandowski

**Affiliations:** Comnovo GmbH, 44263 Dortmund, Germany; ulrich@comnovo.de (S.U.); koester@comnovo.de (V.K.); lewandowski@comnovo.de (A.L.)

**Keywords:** digital twin, collision avoidance, 5G networks, Industry 4.0, safety-critical applications, functional safety, performance level, wireless communication, safety integrity level (SIL), Industrial Internet of Things (IIoT)

## Abstract

This study presents a theoretical framework for defining the performance level of wireless safety functions within industrial environments. While acknowledging the simplifications inherent in our approach—primarily based on packet loss rates as a measure of system performance—the study underscores the dynamic challenges posed by real-world warehouses. Through an in situ measurement study of a forklift truck safety system, we validate the proposed method and emphasize the need for a more nuanced examination of wireless communication in complex settings. The study advocates for an expanded theoretical framework that considers fluctuations in warehouse dynamics, accounting for their impact on packet loss rates and, consequently, the precision of performance-level assessments. Furthermore, the research highlights the complexity introduced by wireless system characteristics not addressed in the simplified model, urging future investigations to incorporate these factors for a comprehensive understanding of wireless safety systems. The absence of specific criteria for wireless systems within existing standards emphasizes the necessity for a specialized framework in addressing safety aspects unique to wireless applications.

## 1. Introduction

Industrial safety functions are critical in preventing accidents, often relying on wired connections to ensure reliability. Redundant connections are employed in high-risk environments to enhance fail-safety. However, in 2022, Germany reported 19,758 industrial truck accidents, eight of which were fatal [1]. In the US, 70 fatal forklift-related accidents were reported [2], highlighting the ongoing challenges in safety.

Truck collisions are a major contributor to accidents, prompting the development of safety functions. While wired communication is the norm due to its perceived reliability, there is a need for innovative solutions, especially for scenarios where wired connections are impractical.

The integration of wireless technologies into safety functions introduces a new dimension, requiring a robust theoretical foundation for assessing their performance level (PL). Existing standards lack specific criteria for wireless communication, creating a gap in safety evaluations. This study addresses this gap by proposing a theoretical approach centered on packet loss rates. Despite inherent simplifications, the approach provides a valuable framework for assessing wireless safety systems.

Our research involves an in situ measurement study in a real industrial environment, focusing on a forklift truck safety system. We validate the theoretical framework through real-world measurements, emphasizing the need for nuanced evaluations in dynamic industrial settings.

The dynamic nature of industrial environments, marked by constant changes in warehouses, challenges the accuracy of simplified metrics. Our study advocates for a more comprehensive framework that considers warehouse dynamics and other influencing factors. This research contributes to the secure implementation of wireless technologies in safety-critical applications within the evolving landscape of Industry 4.0.

In summary, this paper makes a threefold contribution:

- We propose a safety control system for detecting truck collisions.
- We provide real-world measurement results from an industrial site, demonstrating the system’s performance.
- We introduce the first method to determine a PL for wireless safety functions.

The remainder of this paper is structured as follows. In the next section, we present variations of a system for collision avoidance. We then provide traffic measurement results from our in situ study. In Section 4, we discuss how to determine dangerous outages for systems and how to derive a performance level from measurement results. Finally, we discuss our results, what they mean for the industry and what the next steps are.

## 2. Collision Prevention Mechanism

This section explores the literature on collision prevention mechanisms and presents our distinctive system.

### 2.1. Related Work

**Figure 1:**
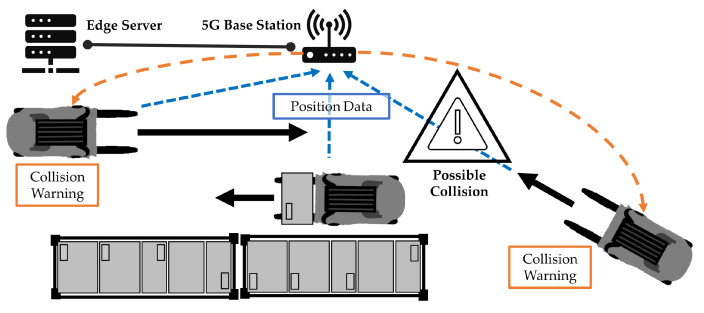
This is an illustration of a potential collision scenario involving two forklift trucks. The black arrows indicate the movement of the trucks. Trucks transmit their position to the base station (blue arrows). The forklift truck on the left is unable to see the oncoming forklift due to a blocked view. An external system uses the positions of the two forklifts to determine the probability for a collision. It may initiate an alarm or send a deceleration command to a vehicle if necessary (orange arrows).

The advancement of the 5G standard, particularly up to 3GPP Release 18, has significantly contributed to the enhancement of applications across various sectors [3]. This progress is particularly beneficial for collision prevention systems, as illustrated in Figure 1. A key application of this technology is the integration of real-time data into digital representations of industrial environments, leading to the creation of ‘digital twins’ of real scenarios. Defined as “a virtual representation of a physical asset, that continuously consumes data from the physical asset, processes it, then provides intelligent feedback to its real counterpart” [4], digital twins play a crucial role in collision prevention by using real-time data on the position and orientation of forklifts. This system not only supports two-way communication, including collision alerts, but also allows the incorporation of additional details such as the forklift’s location and steering direction to create a visual replica of the vehicle. Digital twins are increasingly recognized as effective tools for developing and evaluating complex systems in realistic environments, surpassing traditional simulation methods [4].

A thorough examination of intelligent solutions for enhancing road user safety is presented in the survey by [5]. The survey delves into various collision avoidance systems and ongoing research projects dedicated to applications ensuring the safety of vulnerable road users. The authors explore the potential of leveraging 5G technology to enhance road users’ safety, discussing diverse architectural approaches, such as RAN-wide communication and direct communication between vehicles, pedestrians, or the network. Additionally, they address key performance indicators associated with these solutions. In our research, we also focus on collision avoidance scenarios involving vehicles and pedestrians. However, in contrast to the aforementioned work, our emphasis is on solutions tailored to industrial sites. Notably, our investigation accounts for indoor vehicle traffic, which may not have access to a public RAN.

The authors of [6] give a review of different kinds of collision avoidance systems. They differentiate sensors of such systems into two categories. Active sensors that are used to detect other vehicles include radar, laser, or lidar. Passive sensors collect data without sending sending a signal. They include cameras, microphones, radar and lidar. The review focuses on road safety and thus does not consider limited space indoor scenarios, as in intralogistics. In such scenarios, the difference is that we can establish additional infrastructure which allows for a new range of solutions. Computer vision systems on vehicles can be used to detect other vehicles, pedestrians or other obstacles. In [7], the authors utilize such a system to detect animals that are entering the road, warning the driver or slowing the vehicle. The authors of [8] propose a neural-network-based system that accurately detects and classifies vehicles, achieving about 95% accuracy.

For practical purposes, such systems are very handy if the vehicle already comes equipped with a camera. The advanced in this field are very promising. They are well suited to be used together with an RTLS for increased robustness and reliability. However, high computation power might be necessary, which can lead to high cost. Furthermore, in many industrial areas, there is a high degree of particulate pollution, which requires the cameras to regularly cleaned.

The authors of [9] conduct experiments where robots drive around within a 5G campus network between two cells with their own base station. At the same time, the robots transmit their position in real time to an edge server. In their work, they focus on how the handovers affect the data throughput and the latency and how accurate the positioning system is. Their system shows that RTLS works well over 5G, which is an important part of the system we present in this paper.

The concept of a multi-robot system is explored in [10,11]. Similar to our own system, the authors examine the throughput and round-trip time (RTT) of their system. They suggest a multi-link system with redundant communication, all controlled by a centralized server, which manages various types of robots.

In [12], the authors propose a decentralized collision detection system that is based on proximity detection via UWB signals. They also discuss how their system could be used as a real-time locating system.

### 2.2. System Design

**Figure 2:**
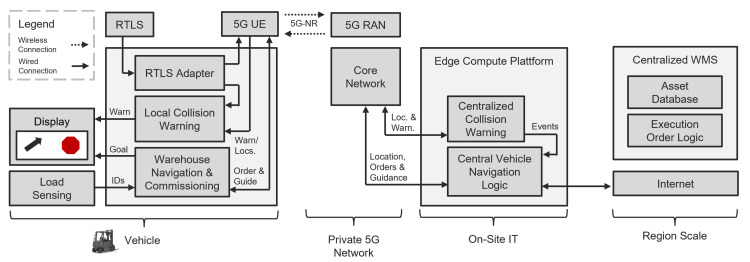
An architecture blueprint from [13] suitable for industrial applications using private 5G networks, showcasing three key elements: the sensor-equipped forklift truck, the local IT services, and the cloud-connected services. Arrows indicate the flow of messages between modules.

**Figure 3:**
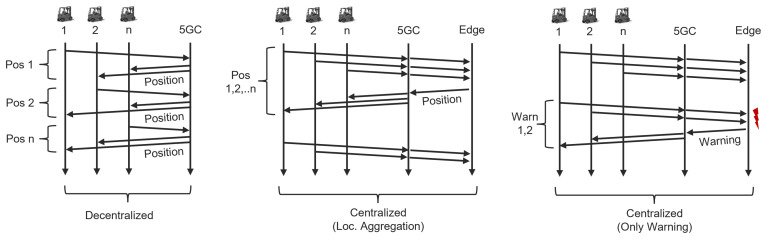
Comparison of communication methodologies among decentralized and centralized systems from [13]. Arrows indicate the flow of packets, similar to a transmission diagram. The icons at the top represent vehicles. The left-side decentralized model uses 5G to disseminate position data among vehicles, which then individually assess collision risk. The middle centralized model involves a server consolidating location data from all trucks and relaying it periodically. The right-side centralized approach calculates potential collisions using incoming location data. The red icon on the right hand side indicates that a collision risk is detected and alerts are sent to all vehicles.

In addition to the architecture depicted in Figure 2, the communication strategy is detailed in Figure 3. The system comprises three primary components: the forklift truck, local IT infrastructure, and cloud-based services with internet connectivity. Beyond collision avoidance, the forklift is fitted with sensors for various applications. An onboard computing unit manages sensor data and controls certain forklift functionalities. The driver receives visual feedback through a display. The computing unit connects with external services, such as Real-Time Location Systems (RTLSs) or edge devices, via a 5G module. This module encompasses a local edge server linked to the 5G network, which is responsible for collating data from forklifts and potentially computing collision scenarios. Additionally, it acts as a gateway to other cloud services or the local network. Enterprise-specific services, like a Warehouse Management System (WMS), are integrated with the local edge server to enhance system capabilities. Internet connectivity allows for the remote monitoring of the logistics system via a web application.

### 2.3. Communication Strategy

As depicted in Figure 3, three methods of collision alerting are outlined. The decentralized model leverages 5G to share positional data among vehicles with each vehicle responsible for assessing collision risk. In one centralized model, the edge server compiles and periodically broadcasts truck locations, easing the 5G network load. Another centralized method utilizes updated truck positions to predict collision events and informs vehicles about potential risks. Centralizing computation reduces the volume of messages in the 5G network, which is a factor that will be further evaluated in the paper.

## 3. Evaluation of Results

This section aims to highlight the potential of a virtual collision avoidance system by comparing its efficiency against traditional methods. Table 1 gives an overview over all variables used in this section.

### 3.1. Experimental Methodology

The designed experiment evaluates the necessity and effectiveness of a 5G-enabled collision warning system in ensuring safety. Initially, the focus is on analyzing the efficiency of various collision avoidance strategies, coupled with a theoretical assessment of 5G network capabilities in handling multiple trucks, based on the proposed communication flow models. Subsequently, a forklift equipped with a 5G-compatible RTLS module is tested within a local campus network. Latency is measured using Internet Control Message Protocol (ICMP) packets to ascertain the system’s efficacy in improving safety in relevant environments.

### 3.2. Efficiency Analysis through Analytical Extrapolation

The efficiency of a virtual collision avoidance system is compared against other methods in an industrial intralogistics setting, involving a fleet of forklifts with a top speed of 15 km/h, which is the common top speed in Europe. Assuming a minimum clear line of sight of 2 m for the driver, a basic braking model is introduced for emergency situations, splitting the total braking distance into reaction and braking distances:

(1) $$d_{b}=\frac{v^{2}}{2a}\;d_{r}=vt_{\mathrm{rct}}\;d_{\mathrm{eb}}=d_{r}+d_{b}$$

**Figure 4:**
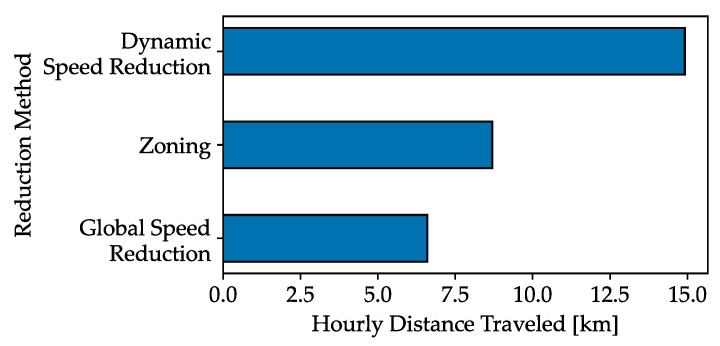
Comparison of distance traveled between collision avoidance methods. Using zoning is shown to be better, while the dynamic braking method is the most efficient.

Here, $d_{r}$ represents the reaction distance, $d_{b}$ represents the braking distance, and $d_{\mathrm{eb}}$ represents the total effective braking distance. The reaction time $t_{\mathrm{rct}}$ is set at 1 s with *v* being the forklift’s speed and *a* the deceleration, which is assumed to be $-10\;m/s^{2}$. The reaction distance is the space needed for the driver to respond to an obstacle. The forklift’s maximum speed is calculated by solving for *v* in the equation with $d_{\mathrm{eb}}=2\;$m, resulting in a top speed of 6.6 km/h. Efficiency is then gauged based on the ratio of drivable distance at this maximum speed, as shown in Figure 4.

This is contrasted with scenarios where trucks are limited to a speed of 6.6 km/h for safety without additional controls. Zoning, another method, restricts speed in areas with limited visibility, with an assumed 25% area limitation. The third method, Dynamic Braking, activates speed reduction in response to detected collision risks. Given the low frequency of collision risks (set at 1%), Figure 4 illustrates that dynamic speed reduction is more efficient than zoning and negates the need for zoning configurations. Therefore, accurate vehicle positioning information, provided by an RTLS via a 5G network, is crucial.

### 3.3. Communication Efficiency Metrics

This section compares the communication flows necessary for effective collision detection. System latency measurements for both centralized and decentralized models, as outlined in Figure 3, are described by

(2) $$\tau_{d}=\frac{1}{f}+\tau_{\mathrm{rtt}}$$

(3) $$\tau_{\mathrm{ca}}=\tau_{\mathrm{cw}}=\frac{1}{f}+\tau_{\mathrm{rtt}}+\tau_{\mathrm{edge}}$$

where $\tau_{d}$ indicates the system latency in decentralized communication, $\tau_{\mathrm{ca}}$ and $\tau_{\mathrm{cw}}$ represent system latency in centralized scenarios with location aggregation and warning-only approaches, respectively. *f* denotes the RTLS rate, $\tau_{\mathrm{rtt}}$ the RTT in the 5G network, and $\tau_{\mathrm{edge}}$ the edge communication and computation delay. The rate of Radio Protocol Data Units (R-PDUs) on the channel is then defined as

(4) $$m_{\mathrm{ca}}=2fn\;m_{\mathrm{cw}}=f\left(n+2P_{w}\right)$$

In this study, we leverage the Network Slicing capabilities of 5G networks for these applications, as discussed in [14,15]. The continuous nature of network traffic for collision warnings necessitates bandwidth management to ensure availability for other critical applications within the local 5G network. With this consideration, we constrain the collision warning application to a maximum bandwidth usage of 2 Mbit/s. For data transmission, we employ the Constrained Application Protocol (CoAP), as outlined in the RFC 7252 specification [16]. CoAP is characterized by its minimal frame size, making it suitable for our bandwidth constraints.

**Figure 5:**
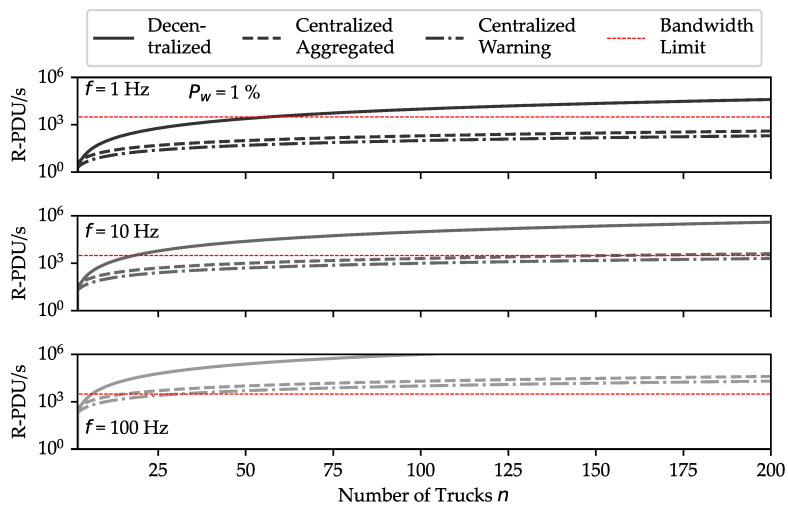
Throughput comparison of messages on the 5G channel for centralized and decentralized models with varying R-PDU packet rates and truck numbers. A 1% warning probability and a 2 Mbit/s bandwidth limitation are assumed. The decentralized model is shown to be less feasible for larger fleets.

As per Table 2, the total packet size relating to the bandwidth limitation gives a maximal amount of $2\;Mbits^{-1}/81\;B\;=3086$ packets per second. Decentralized approaches with numerous trucks face transmission issues not aligned with bandwidth constraints, as demonstrated in Figure 5. Centralization scales transmissions linearly, enhancing performance. Either collision warnings alone can be transmitted, or location information can be selectively sent in dual systems to conserve bandwidth.

### 3.4. Real-World Data Acquisition

**Figure 6:**
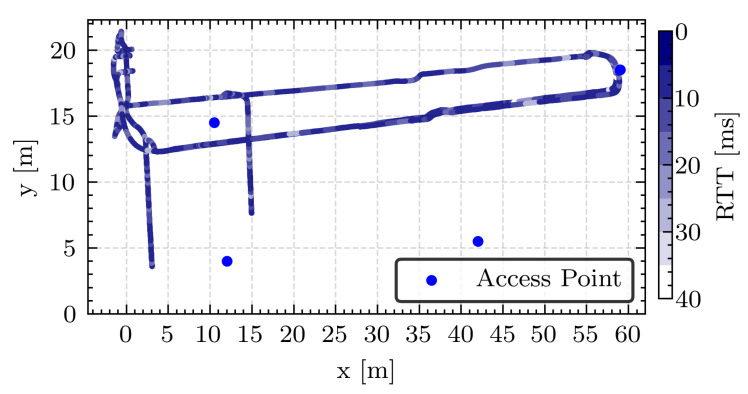
Mapping of forklift position during a test run with the colorscale indicating RTT at specific locations. Four dots represent access points to the local 5G network.

For practical data gathering, an RTLS module was installed on a forklift in an industrial setting. ICMP packet-based RTT measurements were conducted between the forklift’s compute unit and a central control unit connected via a network cable to the 5G core. Figure 6 presents data from one of the test runs, indicating position and RTT. Over 92 min, 5540 data samples were collected at a 1 Hz rate.

**Figure 7:**
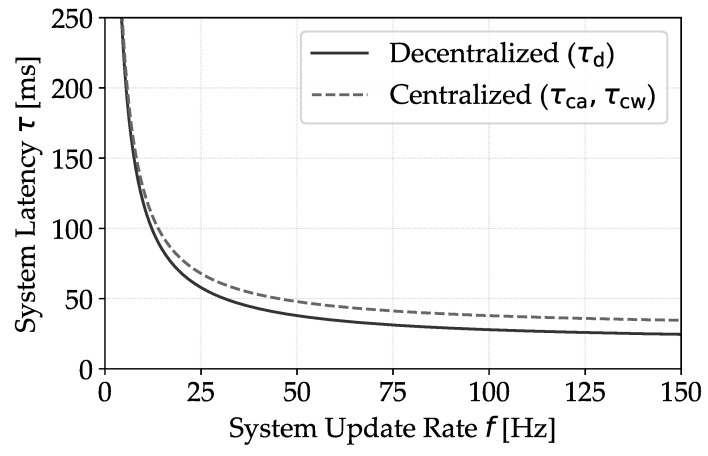
The graph illustrates the interplay between update frequency and delay for the 95th percentile, offering a quantifiable approach for aligning system update rates with delay thresholds to meet the operational standards of collision warning systems.

Figure 7 depicts the interdependence between the system’s update frequency and the associated delay at the 95th percentile. This metric provides a critical benchmark for systems requiring adherence to stringent delay thresholds. Here, $\tau_{\mathrm{edge}}$ is maintained at 10 ms, and the RTT $\tau_{\mathrm{rtt}}$ exhibits a mean of 10.41 ms with a standard deviation of 4.60 ms. The data from the measurements suggest that while the centralized configuration introduces additional latency through edge processing, strategic optimizations in the software and hardware could substantially mitigate this factor. Moreover, by decoupling the truck’s publish rate from the edge server’s update frequency, system efficiency could be enhanced, allowing for less frequent yet more strategic position updates. In situations where real-time position updates are not necessary, a more bandwidth-efficient approach could be used by transmitting updates only when collision events occur. This modification could effectively support the centralized architecture in managing larger fleets, scaling up to larger fleets, according to operational requirements.

### 3.5. Discussion

During our experiments, the total traffic on the system was very low. In the highest load scenario, the network is likely to experience congestion, interference and fading, leading to a much higher latency and packet loss. In a high load scenario, a reserved slot would be recommended for any safety-related communication.

The forklifts that we were using during the experiments had local sensing systems that relied on Lidar and would stop when a person stood in front of it. However, such Lidar systems are very costly and only work locally, which is a major motivation for us to switch to a much cheaper RTLS-based system which in addition works globally. Furthermore, a simple Lidar cannot differentiate between a person, a truck and infrastructure.

## 4. Functional Safety for Wireless Systems

In this section, we discuss functional safety, review existing models for packet loss and discuss the possibility of applying functional safety to wireless systems. The goal of this section is also to provide a tutorial on how to approach the determination of performance level for a safety-function if realized with wireless components.

### 4.1. Background

This section provides background on the functional safety and the reliability of wireless networks.

#### 4.1.1. Functional Safety

Functional safety is defined as “the part of the overall safety of the machine and the machine control system, which depends on the correct functioning of the safety control system and other risk-reducing measures” by the IEC 62061 [17]. The IEC 62061 stands as an international standard developed by the International Electrotechnical Commission (IEC) to guide the design, implementation, and maintenance of safety-related control systems in machinery. Highlighting a holistic approach to the functional safety lifecycle, the standard mandates a thorough risk assessment to identify and evaluate potential hazards associated with machinery. This process facilitates the determination of necessary risk reduction measures. Introducing the concept of performance levels (PLs), expressed as PL *a* to *e*, it quantifies the reliability and performance of safety functions. Aligned with IEC 61508, IEC 62061 employs Safety Integrity Levels (SILs) as a complementary measure. The standard mandates thorough documentation to demonstrate compliance and adherence to functional safety requirements. Given its international nature, IEC 62061 provides a globally recognized framework for addressing functional safety in machinery, making it an essential reference for academic inquiry into safety-related electrical, electronic, and programmable electronic control systems. Transitioning to the realm of wireless systems, the discussion extends to the emerging challenges and considerations in ensuring functional safety in wireless communication within industrial and critical applications.

ISO 13849-1 [18] is a standard that provides guidelines for the design and integration of safety-related control systems in machinery. It introduces the concept of performance levels as a measure of the reliability and performance of safety functions. The performance level is a quantitative measure of the ability of a safety-related control system to perform its safety function under specified conditions. It uses the performance-level classification from PL a (low reliability) to PL e (high reliability). The selection of the appropriate performance level depends on the risk assessment for the specific machinery and its associated safety functions. To assess the risk, three criteria are used [18]. The first criteria is the degree of danger; S1: light reversible injury, S2: serious irreversible injury. The second criteria is the exposition to danger; F1: rare or short exposition to danger, F2: frequent or long exposition to danger. The third criteria is the possibility of danger avoidance; P1: possible under certain circumstances, P1: hardly possible. Table 3 shows which outage frequency is required to reach which PL and SIL.

Often, it is wrongly assumed that a single value, such as the $PFH_{D}$ (Probability of Dangerous Failure per Hour) of a system, is enough to define the performance level of a system. However, this is a misconception. First, performance level is defined for a safety function and not for a system. Second, we must consider the complete chain of systems involved that perform the safety function together. For example, the hardware or the software of the system might also occasionally fail. For a system with *N* safety-related parts of the control system, the total $PFH_{D}$ is determined according to [18] by the sum of each subsystem $PFH_{D}$:

$$PFH_{D}=PFH_{D1}+PFH_{D2}+\cdots +PFH_{DN}$$

Furthermore, there are aspects such as the Mean Time To Dangerous Failure ($MTTF_{D}$) which need to be regarded when assigning a performance level. It is given as

$$\frac{1}{MTTF_{D,\mathrm{total}}}=\sum_{i=1}^{N}\frac{1}{MTTF_{Di}}$$

where $MTTF_{Di}$ is the $MTFF_{D}$ of part *i*. The calculation of $MTTF_{Di}$ is defined in [18].

The German Social Accident Insurance (DGUV) and the Institute for Occupational Safety and Health of the German Social Accident Insurance (IFA) propose the matrix method [19] for the development of safety-related software. It involves a comprehensive risk assessment to identify and mitigate potential hazards in the context of safety-related application software for machinery. It includes defining safety functions and determining performance levels (PLs) based on the assessed risks. Using a matrix, the method correlates safety functions with their required performance levels, aiding in allocating appropriate reliability levels. Specific attention is given to software requirements, encompassing aspects such as architecture, development processes, and validation to achieve the designated PL. They emphasize thorough documentation to record risk assessments, safety functions, and measures taken for maintaining safety integrity.

The absence of a universally accepted performance level (PL) for communication networks stems from the diverse requirements across industries, the dynamic nature of technology, varied risk tolerances, and the complex interdependencies within network ecosystems. Establishing a coherent PL framework necessitates collaborative efforts, beginning with standardization endeavors involving industry stakeholders and organizations. A comprehensive risk assessment framework should be developed, considering the criticality and impact of communication failures in diverse applications. Industry-specific guidelines should be recognized, acknowledging the unique characteristics of different sectors.

To achieve a PL for communication networks, continuous evaluation and adaptation are crucial, reflecting the rapid evolution of technology and the dynamic nature of communication requirements. Any framework should also incorporate cybersecurity considerations, ensuring the security and integrity of network infrastructure. International collaboration is key, engaging with standardization bodies, industry associations, and regulatory authorities to develop universally accepted guidelines. By fostering a collective and adaptive approach, the industry can work toward a standardized performance-level framework that accommodates the diversity and evolving nature of communication networks.

#### 4.1.2. Reliability of Wireless Networks

**Figure 8:**
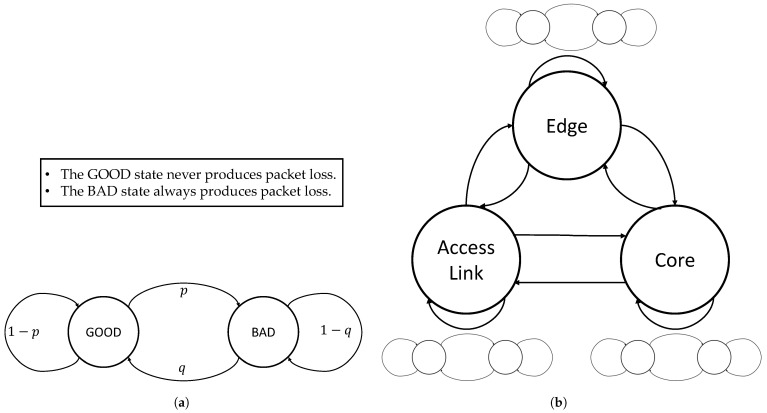
Transition diagrams for Markov chains that model packet loss. (**a**) Simple Gilbert Model (SGM). (**b**) Two-layer SGM/SGM/SGM model. Each outer state contains an SGM (**a**).

For communication networks, loss of packages or messages is the main reason for failure. Therefore, it plays a critical role for the calculation of the performance level of a chain of tools which contain signal transmissions. The Simple Gilbert Model (SGM) [20] is the first important model to describe the burstiness of packet loss and models it as a Markov chain with two states: the GOOD state never produces packet loss; the BAD state always produces packet loss. The transition diagram for the SGM is depicted in Figure 8a. An extension to the SGM as a two-state hidden Markov model (2HMM) is proposed in [21], which is why the SGM and the 2HMM are often referred to as the Gilbert–Elliot model. In this extension, GOOD and BAD have a low/high probability to produce packet loss instead of 0 and 1. However, deriving the hidden state probabilities from measurements is not easy. Furthermore, it performs much worse than the SGM at estimating the max loss burst length and the mean loss burst length [22], which are critical for safety functions. This is why we focus on the SGM in this paper instead. The Extended Gilbert Model (EGM) [23] extends it to n+1 states, where *n* is the number of remembered loss events to describe loss more accurately. In [22], the authors discuss these models for packet loss and argue that they do model packet loss in terms of some metrics for nonbursty traces, but they fail to model packet loss patterns for bursty traces accurately, including SGM and EGM. They propose a complex model that introduces an outer layer to the SGM that consists of three states and that describes the location in the network where the loss event was caused: ACCESS LINK, EDGE, and CORE. The transition probabilities are dependent on the current outer layer state. Furthermore, an SGM may be replaced by above 2HMM in EDGE and CORE for increased accuracy. The transition diagram of the SGM/SGM/SGM variant of their model is depicted in Figure 8b. For the application of safety systems, we would therefore consider using the SGM model for traffic that is only transmitted over the link level and the SGM/SGM/SGM to model packet loss in systems that transmit traffic over the internet.

The study conducted by the authors in [24] investigates the distribution of burst errors and latency in LTE and Wi-Fi. Employing the SGM, they compare the reliability of ultra-reliable channels and redundant LTE and Wi-Fi interfaces. The incorporation of redundant safety channels, as mandated by [18], is crucial for achieving enhanced performance.

In a separate work, the authors of [25] examine packet loss in real-time internet services using the SGM. Their analysis encompasses UDP and TCP streams. A noteworthy contribution of their research lies in proposing a method that extends the SGM to better align with the second-order statistics, thereby improving its applicability to real-world outcomes.

A Kalman filter can also be used to model the packet loss, as shown in in [26]. The authors demonstrate that such a model performs better than the SGM for their real trace, which had 30% packet loss.

The authors of [27] explore strategies aimed at preventing collisions in LoRaWAN channels and assess the impact of unavoidable random access cross-traffic on such strategies. They provide guidelines on how to implement these strategies, introducing a time-scheduled channel access approach. Through simulations, they evaluate the performance of this time-scheduled approach in the presence of access cross-traffic. While LoRaWAN may not be well suited for real-time applications, it finds effective applications in various industrial IoT scenarios. The authors highlight ongoing efforts to enhance its reliability, potentially making it suitable for safety-critical applications where low latency is not a primary concern. The technology’s notable features, including high scalability, extensive coverage, and low power consumption, make it particularly promising for diverse use cases in vast remote areas.

Defining an outage probability for a communication network proves challenging. On the application level, a system may not necessarily experience an outage. However, it is possible to design a system in such a way that brief interruptions in the communication channel can be bridged with heuristics and specific safety mechanisms.

### 4.2. Deriving Performance Level from Measurements

A single package loss event usually does not lead to a failure in the safety function if the next package is transmitted successfully; e.g., when transmitting the position of a truck to another truck for collision avoidance, it is okay if the first package transmission fails as long as the second one is transmitted correctly 100 ms later. It becomes unsafe only when a specific number of consecutive transmissions fail in succession. In the following, the variable *k* represents the number of consecutive package loss events that are required in a system to lead to a failure in the safety function.

A typical network trace might have a loss burst distribution such as that presented in Table 4. If *k* is the critical number of consecutive loss events that leads to an outage, for the example of [23] from Table 4 for k≥4, we would receive a $PFH_{D}=0.00113$. Since $0.00113>10^{-4}$, we cannot even assign PL *a*.

Furthermore, since this is a small sample, let us use the 95% confidence interval given by $\mathrm{CI}=\hat{p}\pm 1.96\sqrt{\frac{\hat{p}\left(1-\hat{p}\right)}{n}}$. For the trace of [23], we receive the interval [0.000492,0.00177]. This means with a likelihood of 95% we can guarantee that the probability of a dangerous outage is at most 0.00177. This would already be too high to assign a performance level.

In our own trace, no losses with k≥4 appeared, since we only measured for an hour and such events are very rare in our system. This demonstrates that just measuring is unreliable, since in safety systems, dangerous outages may only occur a few times per year. This is why, in the following section, we describe how we can use models to derive a good estimate for the frequency of dangerous outages that might not occur during measurement.

### 4.3. Modeling Outage Frequency for Wireless Systems

Safety-related applications are frequently implemented in a fail-safe or redundant way. In the case of a not very time-critical function, this could be realized with a retransmission mechanism. In the case of a time-critical, it is recommended to use redundant transmission over different channels or media, which avoids a possible bursty loss of a single channel. Let us assume a system which transmits a signal four times per second with a uniformly distributed outage probability 1−p=q. We may assume the system to be slightly dangerous (S1) after one second of not receiving a signal ($P\left(X\geq 4\right)=1-\sum_{k=0}^{3}P\left(X=k\right)$) and heavily dangerous (S2) after five seconds of not receiving a signal ($P\left(X\geq 20\right)=1-\sum_{k=0}^{19}P\left(X=k\right)$).

#### 4.3.1. Bernoulli Model for $PFH_{D}$

The probability for each packet to be lost is given as *p*. In a scenario where losing *k* packets in a row would lead to a dangerous situation *D*, the probability for a loss of length *k* is given by [23] as $p_{k}=p^{k-1}\;\cdot \;\left(1-p\right)$ if packets are transmitted with a frequency *f* and we receive the $PFH_{D}$ as follows:

(5) $$PFH_{D}=p^{k}\;\cdot \;\left(1-p\right)\;\cdot \;f\;\cdot \;3600s$$

For a safety function, we assume that there is a duration *T*, so that the system is no longer safe if an outage with duration ≥T occurs. Since it is k=T·f, for a system with frequency *f*, we can generalize the equation to

(6) $$PFH_{D}=p^{T\;\cdot \;f}\;\cdot \;\left(1-p\right)\;\cdot \;f\;\cdot \;3600s$$

**Figure 9:**
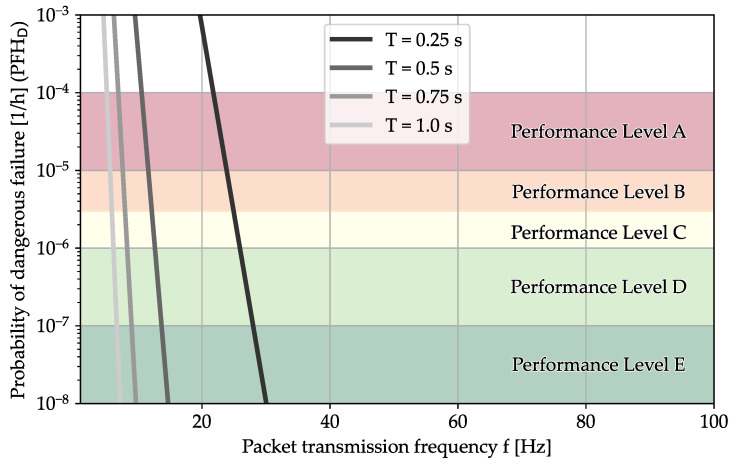
Reachable performance level with 1% packet loss for various packet transmission rates *f* and times to dangerous with outage *T*, assuming a uniform distribution. This model disregards the bursty behavior of packet loss and is only recommended for nonbursty loss of data.

Let us consider a scenario where the wireless communication has a probability of 99% to transmit a message successfully. For this scenario, we assume that a safety function prevents accidents. If it does not receive the messages in time *T*, the safety function fails and a dangerous accident can no longer be prevented. In Figure 9, the x-axis represents the frequency with which messages are sent. The y-axis represents the probability of a dangerous situation arising. The colored areas in the plot show the reached performance level. In this case, we can see that in a scenario where T=1s, we need a message frequency of more than 5Hz to reach PL A and more than 6Hz to reach PL D.

Given that PL pertains to a specific safety function, it is essential to ensure its efficacy precisely at the point of application. While this requirement is negligible for wired connections, where signals behave uniformly, it is crucial for wireless signals due to environmental variations. In wireless scenarios, factors such as obstacles between the sender and receiver can significantly affect transmission. Therefore, to guarantee a specific PL, it is necessary to measure the outage probability for each location where the function is employed.

However, in this paper we mainly consider vehicular applications where sensors might also be applied to vehicles, especially forklift trucks. In industrial areas, forklift trucks are used to stack large objects which then block the line of sight, weakening the signal and making it more error prone. So, the signal is always variable.

#### 4.3.2. SGM for $PFH_{D}$

A common use of a safety functions is the direct transmission of data between local machines without routing it via the internet. For packet loss on the access link, the SGM model has proven to be accurate [22]. Using it, we can calculate *p* and *q* from measurements for the SGM, given $m_{k}$ represents the amount of observed loss events of length *k*.

$$p=\frac{\sum_{k=1}^{n-1}m_{k}}{m_{0}}$$

$$q=1-\frac{\sum_{k=2}^{n-1}m_{k}\;\cdot \;\left(k-1\right)}{\sum_{k=1}^{n-1}m_{k}\;\cdot \;k}$$

For our data set from Section 3, this results in p=0.044,q=0.895, which can also be written as

$$\mathrm{SGM}=\left[\begin{array}{cc} 0.956 & 0.044\\ 0.895 & 0.105 \end{array}\right]$$

Furthermore, with $p_{k}={\left(1-q\right)}^{k-1}\;\cdot \;q$, given k=T·f, we receive the loss distribution in Table 5. We then substitute *p* and *k* in Equation (6) with 1−q and T·f and normalize it to events/hour.

(7) $$PFH_{D}={\left(1-q\right)}^{\left(T\;\cdot \;f-1\right)}\;\cdot \;q\;\cdot \;f\;\cdot \;3600s$$

**Figure 10:**
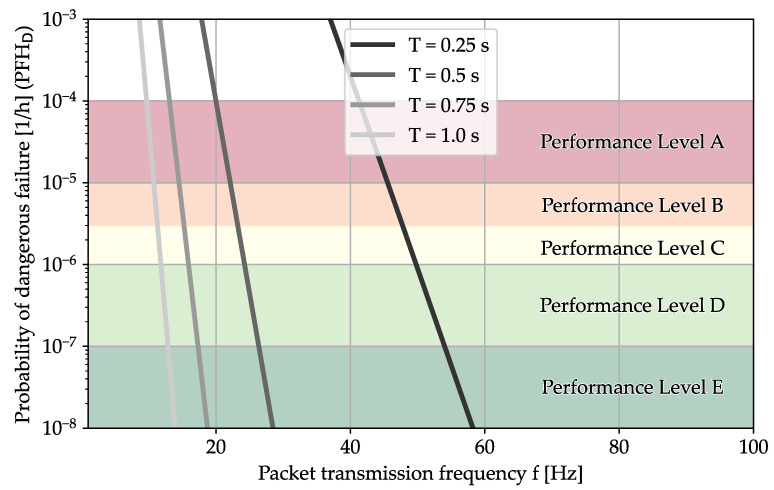
With the SGM, we receive the following distribution for our measurements. This is the recommended model for traffic only flowing through the access link.

In Figure 10, we see the effect of the packet transmission frequency *f* and the critical outage duration *T* on the reachable performance level if we apply the SGM to our measurements. We see that given our own case, where we consider the limit for outages at T=1s, we need T=10Hz for performance level A and T=12Hz for D.

#### 4.3.3. Two-Layer SGM/SGM/SGM for $PFH_{D}$

In this section, we consider the two-layer SGM/SGM/SGM model from [22] for the case of an internet-based safety system. For the outer states, for the edge and for the core, we use the numbers from the example bursty trace from [22]. For the access link, we use our own results from above.

$$\begin{array}{cc} \mathrm{Outer}\;\mathrm{states} & =\left[\begin{array}{ccc} 0.9263 & 0 & 0.0632\\ 0 & 0 & 0\\ 0.1182 & 0 & 0.8818 \end{array}\right]\\ \mathrm{Access}\;\mathrm{Link}\;\mathrm{SGM} & =\left[\begin{array}{cc} 0.956 & 0.044\\ 0.895 & 0.105 \end{array}\right]\\ \mathrm{Edge}\;\mathrm{SGM} & =\left[\begin{array}{cc} 1 & 0\\ 0 & 1 \end{array}\right]\\ \mathrm{Core}\;\mathrm{SGM} & =\left[\begin{array}{cc} 0.9682 & 0.0318\\ 0.9071 & 0.0929 \end{array}\right] \end{array}$$

We simulated this Markov chain with $10^{7}$ events, resulting in the burst loss count in Table 6. We derive $PFH_{D}$ by fitting the data and normalizing the resulting equation to

$$PFH_{D}=3.38\;\cdot \;10^{-3}\;\cdot \;e^{-T\;\cdot \;f}\;\cdot \;f\;\cdot \;3600$$

**Figure 11:**
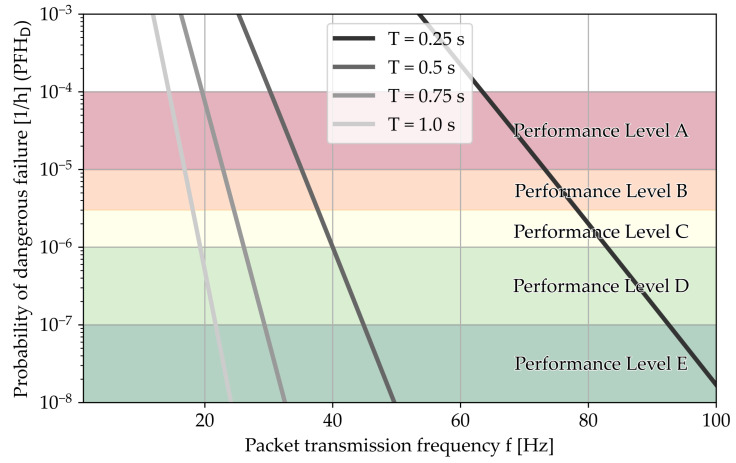
With the SGM/SGM/SGM model, we receive the following distribution. This model is recommended for safety systems that transmit data over the internet.

Figure 11 shows us that with T=1s, we would need T=14Hz for performance level A and T=19Hz for D. The additional possibility for loss on the core leads to a significant increase in total loss, which is why we would need a higher packet transmission frequency to reach the same $PFH_{D}$ in a scenario which is otherwise unchanged.

### 4.4. Strengths and Limitations of the Study

Our study presents both strengths and limitations in our approach to defining the PL of wireless safety functions in industrial settings.

Regarding the novelty of our approach, our research introduces a pioneering theoretical framework that fills a crucial gap in existing safety evaluations. By developing this framework, we provide industries with a valuable tool to assess the effectiveness and reliability of wireless safety systems. Moreover, our framework is not just theoretical; it has been validated through real-world measurements in industrial environments, demonstrating its practical applicability and effectiveness in ensuring safety.

Additionally, our study identifies a significant gap in existing standards regarding the evaluation of wireless safety functions. By highlighting this gap, we pave the way for the development of dedicated criteria tailored specifically to address the unique challenges posed by wireless communication in safety-critical applications. This recognition of the gap underscores the importance of our research in guiding future standards and regulations in the field.

However, our study is not without its limitations. We acknowledge that our approach simplifies system performance assessment by primarily focusing on packet loss rates. While this provides a useful quantitative measure, it may overlook other critical factors that influence system reliability. Furthermore, our method may not fully capture the dynamic nature of real-world industrial environments, such as fluctuations in warehouse conditions, which can impact the accuracy of our performance-level assessments.

In summary, our study offers a valuable contribution to the field of wireless safety systems by providing a novel theoretical framework and identifying gaps in existing standards. While our approach has its limitations, it lays the foundation for further research and development in ensuring the safe and reliable operation of wireless systems in industrial settings.

## 5. Discussion

In conclusion, this study introduces a theoretical approach to define the performance level of wireless safety functions in industrial settings. Nevertheless, it is crucial to recognize the inherent simplifications in our method, as it assumes that packet loss rates are a primary measure of system performance. The dynamic nature of real-world warehouses, e.g., constant changes such as goods rearrangement, challenges the accuracy of this simplified metric.

Our in situ measurement study, validating the proposed method in a practical safety system for forklift trucks, underscores the necessity for a more nuanced examination of wireless communication in complex environments. While the theoretical framework holds value, it should be expanded to account for the dynamic nature of warehouses, accommodating fluctuations that may impact packet loss rates and, consequently, the precision of performance-level assessments.

Furthermore, specific wireless system characteristics, including factors like reflections not addressed in our simplified model, add complexity to the real-world performance of collision avoidance systems. Future research should broaden the evaluation scope to include these additional influencing factors for a more comprehensive understanding of wireless safety systems. Additionally, it is crucial to recognize that reliability assessments should not be confined solely to burst-like packet loss scenarios. In safety-critical applications, factors such as positioning inaccuracies and varying packet loss rates, not necessarily stemming from burst events, must be thoroughly investigated. These factors, integral to the real-world complexities of industrial environments, significantly influence the overall performance and safety of communication networks.

In summary, our exploration reveals that prominent standards such as ISO IEC 62061 [17], ISO 13849-1 [18], 26262 [28], and IEC 61508 [29] generally lack specific criteria tailored to wireless systems. These standards predominantly emphasize the functional safety of electrical, electronic, and programmable electronic systems without explicitly addressing the unique considerations of wireless communication. To date, there have only been a few examples of safety-oriented radio systems, such as remote controls for cranes, which are typically specified to performance level C (cf. EN 13849-1). However, in these cases, the functionality often limits itself to the “stop” condition as a safe state. In the context of collision avoidance systems, though, such a state is not feasible. In these systems, vehicles must remain maneuverable in the event of an incident and cannot transition into a complete stop.

The lack of specific guidelines for wireless systems within existing norms emphasizes the necessity for a dedicated framework to thoroughly evaluate safety aspects in wireless applications. With industries incorporating wireless technologies into safety-critical processes, there is an escalating need to establish precise criteria and guidelines tailored to address the unique challenges presented by wireless communication.

This study establishes the groundwork for further research endeavors aiming to bridge the gap between theoretical models and the intricate realities of industrial environments. Enhancing the method to incorporate the dynamics of warehouses and considering broader influencing factors will contribute to more practical and robust conclusions, facilitating the secure implementation of wireless technologies in safety-critical applications within the evolving landscape of Industry 4.0.

## Figures and Tables

**Table 1:** Overview of variables.

Variable	Description	Unit
*v*	Forklift speed	km/h
*a*	Deceleration	m/s^2^
$d_{b}$	Braking distance	m
$d_{r}$	Reaction distance	m
$d_{\mathrm{eb}}$	Total effective braking distance	m
$t_{\mathrm{rct}}$	Reaction time	s
$\tau_{d}$	Maximum delay in decentralized communication	ms
$\tau_{\mathrm{ca}}$	System latency in centralized scenario with location aggregation	ms
$\tau_{\mathrm{cw}}$	System latency in centralized scenario with warning-only approach	ms
*f*	RTLS rate	Hz
$\tau_{\mathrm{rtt}}$	Round-trip time in the 5G network	ms
$\tau_{\mathrm{edge}}$	Edge communication and computation delay	ms
$m_{\mathrm{ca}}$	Number of Radio Protocol Data Units (R-PDUs) on the channel for centralized with location aggregation	-
$m_{\mathrm{cw}}$	Number of R-PDUs on the channel for centralized with warning-only approach	-
$P_{w}$	Probability of warning	%
*n*	Number of trucks	-

**Table 2:** Byte composition of a real-time location system (RTLS) packet.

Component	Size
IPv6 Header	40 B
UDP Header	8 B
CoAP Header	9 B
Payload (Position, Orientation, Speed, Timestamp)	24 B
Total Packet Size	81 B

**Table 3:** Relationship between Performance Level (PL), mean probability, and Safety Integrity Level (SIL).

PL	Mean Probability [1/h]	SIL
a	$\geq 10^{-5}$ to $<10^{-4}$	-
b	$\geq 3\times 10^{-6}$ to $<10^{-5}$	1
c	$\geq 10^{-6}$ to $<3\times 10^{-6}$	1
d	$\geq 10^{-7}$ to $<10^{-6}$	2
e	$\geq 10^{-8}$ to $<10^{-7}$	3

**Table 4:** Burst loss count for two data sets.

Burst Length *k*	0	1	2	3	4	5
Trace [23] Count $m_{k}$	9992	469	144	24	7	5
Trace Section 3 Count $m_{k}$	107,558	203	8	1	0	0

**Table 5:** Burst loss distribution of our data set according to SGM.

Burst Length *k*	1	2	3	4	5	6	7	8	9	10
Probability $p_{k}$	0.895	0.094	0.0099	0.001	$1.09\times 10^{-4}$	$1.1\times 10^{-5}$	$1.2\times 10^{-6}$	$1.3\times 10^{-7}$	$1.3\times 10^{-8}$	$1.4\times 10^{-9}$

**Table 6:** Burst loss count of our data set according to SGM/SGM/SGM.

Burst Length	0	1	2	3	4	5	6
Count	$9.21\times 10^{6}$	$3.38\times 10^{5}$	$3.40\times 10^{4}$	$3.44\times 10^{3}$	383	39	4

## Data Availability

The data presented in this study are available on request from the corresponding author. The data are not publicly available due to being measured at a private industrial site, and we do not have permission from the corresponding private company to publish them.

## References

[1] DGUV (2022). Statistik—Arbeitsunfallgeschehen 2022.

[2] Forklift-Related Injuries, United States, 2011–2021. https://injuryfacts.nsc.org/work/safety-topics/forklifts/.

[3] Lin X. (2022). An overview of 5G advanced evolution in 3GPP release 18. IEEE Commun. Stand. Mag..

[4] Mihai S., Yaqoob M., Hung D.V., Davis W., Towakel P., Raza M., Karamanoglu M., Barn B., Shetve D., Prasad R.V. (2022). Digital twins: A survey on enabling technologies, challenges, trends and future prospects. IEEE Commun. Surv. Tutor..

[5] Zoghlami C., Kacimi R., Dhaou R. (2023). 5G-enabled V2X communications for vulnerable road users safety applications: A review. Wirel. Netw..

[6] Mukhtar A., Xia L., Tang T.B. (2015). Vehicle detection techniques for collision avoidance systems: A review. IEEE Trans. Intell. Transp. Syst..

[7] Sharma S.U., Shah D.J. (2016). A practical animal detection and collision avoidance system using computer vision technique. IEEE Access.

[8] Sang J., Wu Z., Guo P., Hu H., Xiang H., Zhang Q., Cai B. (2018). An improved YOLOv2 for vehicle detection. Sensors.

[9] Luong T., Barros G., Boshoff M., Moldovan C., Schuster D., Gruhn V., Kuhlenkötter B. Investigating the 5G Handover in Autonomous Mobile Robotic Applications. Proceedings of the 3rd International Conference on Robotics, Automation, and Artificial Intelligence (RAAI 2023).

[10] Patchou M., Gebauer T., Krieger C., Böcker S., Wietfeld C. Distributed Realtime Wireless Network Emulation for Multi-Robot and Multi-Link Setup Evaluation. Proceedings of the 2023 IEEE International Conference on Safety, Security, and Rescue Robotics (SSRR).

[11] Arendt C., Patchou M., Böcker S., Tiemann J., Wietfeld C. Pushing the limits: Resilience testing for mission-critical machine-type communication. Proceedings of the 2021 IEEE 94th Vehicular Technology Conference (VTC2021-Fall).

[12] Mastrolembo Ventura S., Bellagente P., Rinaldi S., Flammini A., Ciribini A.L. (2023). Enhancing Safety on Construction Sites: A UWB-Based Proximity Warning System Ensuring GDPR Compliance to Prevent Collision Hazards. Sensors.

[13] Ulrich S., Luong T., Moldovan C., Tiemann J., Lewandowski A., Röhrig C. System Architecture for Digital Twin based Collision Avoidance through Private 5G Networks. Proceedings of the 2023 IEEE 12th International Conference on Intelligent Data Acquisition and Advanced Computing Systems: Technology and Applications (IDAACS).

[14] Bektas C., Böcker S., Kurtz F., Wietfeld C. Reliable Software-Defined RAN Network Slicing for Mission-Critical 5G Communication Networks. Proceedings of the 2019 IEEE Globecom Workshops (GC Wkshps).

[15] Kurtz F., Bektas C., Dorsch N., Wietfeld C. Network slicing for critical communications in shared 5G infrastructures-an empirical evaluation. Proceedings of the 2018 4th IEEE Conference on Network Softwarization and Workshops (NetSoft).

[16] Shelby Z., Hartke K., Bormann C. (2014). The Constrained Application Protocol (CoAP).

[17] International Electrotechnical Commission (2005). Safety of Machinery—Functional Safety of Safety-Related Electrical, Electronic and Programmable Electronic Control Systems.

[18] (2023). Safety of Machinery—Safety-Related Parts of Control Systems—Part 1: General Principles for Design.

[19] Huelke M., Becker N., Eggeling M. (2016). Sicherheitsbezogene Anwendungssoftware von Maschinen—Die Matrixmethode des IFA.

[20] Gilbert E.N. (1960). Capacity of a burst-noise channel. Bell Syst. Tech. J..

[21] Elliott E.O. (1963). Estimates of error rates for codes on burst-noise channels. Bell Syst. Tech. J..

[22] Ellis M., Pezaros D.P., Kypraios T., Perkins C. (2014). A two-level Markov model for packet loss in UDP/IP-based real-time video applications targeting residential users. Comput. Netw..

[23] Jiang W., Schulzrinne H. Modeling of packet loss and delay and their effect on real-time multimedia service quality. Proceedings of the NOSSDAV.

[24] Nielsen J.J., Leyva-Mayorga I., Popovski P. Reliability and error burst length analysis of wireless multi-connectivity. Proceedings of the 2019 16th International Symposium on Wireless Communication Systems (ISWCS).

[25] Haßlinger G., Hohlfeld O. The Gilbert-Elliott model for packet loss in real time services on the Internet. Proceedings of the 14th GI/ITG Conference-Measurement, Modelling and Evalutation of Computer and Communication Systems, VDE.

[26] Silva C.A.G.D., Santos E.L.D. (2023). A Compensation Model for Packet Loss Using Kalman Filter in Wireless Network Control Systems. Energies.

[27] Loh F., Mehling N., Hoßfeld T. (2022). Towards LoRaWAN without Data Loss: Studying the Performance of Different Channel Access Approaches. Sensors.

[28] (2011). Road vehicles—Functional Safety.

[29] (2000). Functional Safety of Electrical/Electronic/Programmable Electronic Safety Related Systems.

